# Unfamiliar Handler-Induced Hypercortisolism, Abnormal Oral Behaviors, and Delayed Inflammatory Response in Pre-Weaned Zebu Cross Calves

**DOI:** 10.3390/ani16121795

**Published:** 2026-06-10

**Authors:** María de la Luz Barrios-Moreno, Elein Hernandez, Víctor Manuel Díaz Sánchez, Elke von Son-de Fernex

**Affiliations:** 1 Teaching, Research and Extension in Tropical Livestock Center, Faculty of Veterinary Medicine and Zootechnics, National Autonomous University of Mexico, Martínez de la Torre 93600, Mexico; mariabarriosm02@gmail.com; 2 Faculty of Higher Studies Cuautitlán, National Autonomous University of Mexico, Km 2.5 Carretera Cuautitlán-Teoloyuca, Cuautitlán Izcalli 54714, Mexico; victordiaz@cuautitlan.unam.mx

**Keywords:** cattle welfare, cortisol, c-reactive protein, handler, tropical livestock, zebu cattle

## Abstract

The physiological impact of climatic stress is well documented in ruminants, but the acute stress resulting from personnel rotation and its health and welfare consequences are poorly understood. The objective of this observational prospective longitudinal study was to assess the endocrine, inflammatory, behavioral, and cardiorespiratory responses of pre-weaned Zebu-cross calves (¾ Zebu x ¼ Holstein) to handler substitution and environmental variables in the Mexican humid tropics during the rainy season. Fourteen calves were monitored over a 68-day period. The initial behavioral (n = 14) and physiological (n = 5) assessments (S1) were conducted after a 30-day stabilization period with the familiar handler to ensure that animals had reached a physiological steady state, free from the influence of previous personnel rotations and from familiarization with two external observers, prior to the first assessment. Subsequently, the handler was replaced for 17 days (S2). The third (S3) and fourth (S4) samplings were conducted after the familiar handler was reinstated (21-day). There was a significant increase in plasma cortisol and abnormal suckling behaviors at S2 compared to S1 and S4, and a C-reactive protein increase in S3. Furthermore, strong correlations were found between calf handler and cortisol, heart rate, respiratory rate, latency to suckle, and cross-sucking, suggesting an acute stress response with a delayed inflammatory response secondary to the handler’s substitution. Environmentally, ambient temperature (AT, °C) showed no correlation with cortisol levels, indicating that the primary driver of the neuroendocrine and behavioral changes was handler substitution rather than climatic variations.

## Simple Summary

In tropical livestock systems, animal welfare is often compromised by environmental and management factors. The human-ruminant relationship (HRr) is a cornerstone of livestock welfare, yet the physiological consequences of breaking this bond are frequently overlooked, particularly in crossbred zebu calves. In this study, a single acute stress event induced by an unfamiliar calf handler elicited an acute stress response, evidenced by increased plasma cortisol, the onset of abnormal suckling behaviors, and changes in heart rate and other metabolic parameters, with a delayed inflammatory response. These findings suggest that the calf handler identity is a potent acute stressor for calves, emphasizing the need for consistent management routines that minimize welfare risks and do not compromise the immunological response of young animals. Ensuring consistency among calf handlers is, therefore, a vital management strategy for optimizing animal welfare in tropical cattle production units.

## 1. Introduction

Previous studies have focused on the positive human–cattle relationship and its impact on productivity, personnel perceptions, and animal behavior and welfare [1,2,3]. However, most animal welfare assessments of cattle are based on European cattle and intensive production systems [4,5]. Animal welfare assessment in Zebu cattle (*Bos indicus*) and zebu-crossbred cattle is relevant because they are raised under extensive conditions and are known to be more reactive to handling than European cattle [6,7]. To our knowledge, research on zebu cattle behavior has mainly focused on genetic factors, maternal behavior, and some adult cattle handling practices, but much remains to be understood about calves’ behavior and its relationship to their handling [6,7,8,9].

Stress has been defined as an alteration of the body’s homeostasis, originating from real or perceived internal or external adverse effects (stressors), that impacts calves’ health, welfare, and performance [10]. The primary response to stressful stimuli is activation of the hypothalamic–pituitary–adrenal (HPA) axis, leading to cortisol release. Although it is essential for acute stress adaptation, it becomes detrimental when secretion is prolonged (hypercortisolism). Hypercortisolism triggers a systemic metabolic shift in claves promoting gluconeogenesis by mobilizing amino acids from muscular tissue and inhibiting glucose uptake in peripheral tissues to prioritize energy for vital organs. In the tropical context, this increased metabolic rate is particularly detrimental, as it generates endogenous heat, potentially pushing calves toward a secondary thermal shock if there is deviation from the thermoneutral zone for zebu calves (18.15 and 30.10 °C) [11]. Furthermore, chronic elevation of cortisol levels in pre-weaned calves (above 25–30 ng/mL) leads to thymus involution, leading to immunosuppression (lymphopenia and neutrophilia) and altering the respiratory mucosa, creating an immunological window of vulnerability that can persist for 48 to 72 h, favoring pathogen colonization [12]. Also, stressed calves decrease dry matter intake and, consequently, average daily gains and carcass quality [10]. Some of the main stressors reported to trigger significant HPA include: (i) anthropogenic and social stressors (unfamiliar handlers, abrupt weaning), (ii) physical and management (aversive handling, painful procedures, transport), (iii) environmental stressors (heat stress).

Cattle are social animals, and handlers must be familiar with situational awareness in cattle, which includes the handler’s continuous assessment of the environment and interpretation of environmental and animal changes to prevent issues or take action to ensure animal welfare [13]. Nonetheless, this vital relationship between animals and handlers could put animal welfare at risk due to the handler’s experience, personnel rotation, or stress secondary to other management practices (e.g., calving, milking, dehorning, etc.), as in other breeds [8,13]. Traditionally, calf handlers have been viewed merely as food providers or health inspectors, rather than central figures in the animal’s social environment. However, recent research has proven that ruminants are not passive receptors; they develop social memory and cognitive representations of their environment, management, and handlers [14]. Calves depend directly on the care provided by human handlers, and management failures increase morbidity and mortality rates, with both ethical and economic implications [15]. These management failures can be secondary to employee turnover, resulting in inconsistencies in animal care and production efficiency [16,17].

In Latin America, it has been reported that calf management practices have a direct impact on morbidity and mortality, increasing the rates up to 33–50% and 13.7%, respectively [18,19,20]. Poor practices frequently induce acute stress, triggering cortisol spikes that compromise the immune system. This creates a window of vulnerability that exposes calves to pathogens. Specifically, personnel rotation and handler substitution in pre-weaned calves can induce acute stress responses, behavioral changes such as coping mechanisms, and delayed inflammatory responses. Thus, the objective of this study was to assess the endocrine, inflammatory, cardiorespiratory, and behavioral responses of pre-weaned Zebu-cross calves (¾ Zebu x ¼ Holstein) to handler substitution and environmental variables in the Mexican humid tropics.

## 2. Materials and Methods

### 2.1. Study Area

This research work was carried out from July to September 2025 at the Teaching, Research, and Extension in Tropical Livestock Center (CEIEGT), Faculty of Veterinary Medicine and Zootechnics, National Autonomous University of Mexico (FMVZ-UNAM), Martínez de la Torre, Veracruz, Mexico (lat: 20.036, lon: −97.105; 151 m above sea level), featuring a mean ambient temperature of 31.88 ± 0.79 °C and a daily rainfall range of 0.91–11.85 mm during the sampling periods. Daily ambient temperature (AT, °C) was determined using a digital thermohydrometer (HTC-2, Edalme^®^, Edo. Mex, Mexico) while average daily rainfall (AR, mm) was obtained from National Aeronautics and Space Administration (NASA) Langley Research Center’s Prediction Of Worldwide Energy Resources (POWER) project funded through the NASA Earth Science Division. From the POWER Project’s Hourly v2.6.2 on 28 April 2026 (lat: 20.036, lon: −97.105, NASA POWER I DAV v2.6.2) [21]. The study was conducted in accordance with the Internal Committee for the Care and Use of Animals (CICUA, in Spanish) from the FMVZ-UNAM (Approved Animal Care and Use Protocol CICUA-2024/170).

### 2.2. Calves and Animal Management

**Figure 1:**
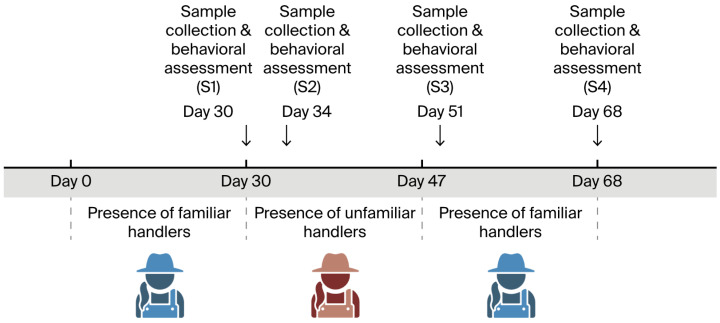
Timeline of experimental methodology for handler substitution in Zebu cross calves. Four time points were used for blood sample collection, physical exams, and behavior assessment recording (S1, S2, S3, S4). The calves were initially managed during artificial lactation by the same handlers for 30 days. The first sampling (S1) occurred at the end of this period. The handler was replaced with an unfamiliar handler for 17 days, and an experimental sampling was conducted on the 34th day (S2). The familiar handler returned on the 48th day of the experiment, and two more samplings were conducted (S3 and S4). The graphical elements were created with BioRender.com. We thank BioRender for providing the scientific icons used in this illustration.

The calves assessed in this observational prospective longitudinal study were monitored without interfering with the regular management practices of the production unit. Fourteen crossbred calves (¾ Zebu x ¼ Ho), two months old, with individual ear tags, were reared under a tropical rearing system with artificial lactation, managed twice daily by a single handler who alternates on weekends with another handler (also familiar). Lactation was performed using a six-station calf feeder with rubber nipples, and calves were allowed access in groups of six. Whole milk was transported daily from the milking parlor to the calf rearing area and offered at an average temperature of 38 °C. The calves were fed 2 L of whole milk twice a day at 9:00 h and 13:00 h. Upon completion, all six were transferred into two interconnected pens of 25.47 m^2^ and 24.98 m^2^, equipped with trough feeders, drinking troughs and a roof, where all calves were complemented with 1 kg of commercial concentrate per calf (Protein 20% 5 min, fat 2% min, fiber 8% max, humidity 12% max, ash 8% max, Nitrogen-Free extract 50%). After the liquid diet and concentrate supplementation, all calves shared a grazing area (4569 m^2^) divided into three paddocks where pasture rotation is used to optimize the use of grass and space. All the calves had free access to clean water and a shelter pen. Using a computer true random number generator (random.org), calves were numbered from 1 through 14 for identification and monitored over a 68-day period. The handlers’ rotation management and sampling periods are shown in Figure 1.

All handlers were experienced farm staff with several years of hands-on work and prior training to carry out their daily duties. The familiar handlers have over 9 years of experience in calf rearing, while the unfamiliar handlers assist with farm activities as needed. They were blinded to the objective of the experiment. The familiar handlers were replaced at S2 due to the workers’ contractual 17-day vacation. During this time-lapse, calves were managed by two unfamiliar handlers; no further changes were made during this 68-day trial.

From an ethical standpoint and in accordance with the 3Rs approaches (Replacement, Reduction, and Refinement of animals in research) as mandated by our own institution committee CICUA-FMVZ-UNAM, sampling a subset of 5 calves for minimally invasive blood collection, as a pilot study, while monitoring all 14 non-invasively for behavior, was an ethical choice to minimize group distress while robustly validating our biological hypothesis. This cohort size (n = 5) proved to be fully optimized for detecting physiological responses. For instance, the high rank-sum difference observed in cortisol levels (13.00 out of a maximum possible 15 for S2 vs. S4), demonstrates a highly homogeneous neuroendocrine response among calves. Furthermore, the results from the Spearman rank correlation matrix indicate that sampling a subset of n = 5 calves provides sufficient statistical power to accurately detect and correlate systemic and behavioral shifts. Thus, from the population of 14 calves, five were selected using a computer true random number generator provided by Haahr [22] for subsequent blood sampling to evaluate physiological stress indicators.

### 2.3. Physiological and Metabolic Assessment

The five preselected calves were handled and physically restrained as described in standard operating procedures for blood collection in cattle [13,14], and all procedures were performed by a licensed veterinarian. Simultaneously, a routine medical check-up was performed, during which heart rate (HR), respiratory rate (RR), and rectal temperature (BT) were recorded, along with any other incidental findings. During each sampling period (S1–S4), physiological stress indicators, including serum cortisol (CORT), C-reactive protein (C-RP), and metabolic markers (BUN, Urea, Glucose, and Creatinine), were recorded. Blood samples were taken at 7:00 h directly from the jugular vein using 10 mL vacutainers (with and without EDTA) and sent to a local certified veterinary clinical laboratory (ELÍ -Laboratorio Veterinario, Martínez de la Torre, Veracruz, Mexico) for further processing. Serum samples were processed in the lab for cortisol concentration determined by a competitive immunoluminometric assay (MAGLUMI™ 800, Snibe, Shenzhen, China), while the determination of C-reactive protein was done with a slide agglutination test (CRP-latex method) using a KJ-201BD Horizontal Shaker Oscillator (Jiangsu Kangjian Medical Apparatus Co., Ltd., Taizhou, China). Glucose, blood urea nitrogen (BUN), and creatinine (CREAT) were measured using a dry chemistry analyzer (DRI-CHEM NX500i, Fujifilm Co., Tokio, Japan), while urea was determined by arithmetic calculation.

### 2.4. Behavior Assessment

Behavioral assessments of all 14 calves were conducted through direct on-site observations during lactation in the six-station calf feeder and in the two interconnected pens used for feed supplementation. Video recordings were taken by hand on a mobile phone (iPhone 14, Apple, Cupertino, CA, USA) for subsequent analysis. Behavioral variables (Table 1) were evaluated from the moment calves were given access to the six-station calf feeder until the last calf finished eating, as well as during their subsequent concentrate supplementation in the pen. Observations were recorded during both the morning and afternoon feedings. One trained observer assessed the calves’ behavior using the ethogram in Table 1, adapted from reported suckling behaviors in calves [23,24]. The video recording included only the animals, with the handler out of frame to avoid bias during later assessment. The observer was blinded to the specific sampling time and was not present during the recording, but differences in the calves’ normal growth between samplings are unavoidable.

### 2.5. Statistical Analysis

All physiological and behavioral data were analyzed using GraphPad Prism software (version 11.0.1 (99)). Cortisol (CORT), C-reactive protein (C-RP) and Heart rate (HR) were analyzed using the non-parametric Friedman test. Glucose, creatinine, urea and blood urea nitrogen, body temperature, and respiratory rate were analyzed using a RM One-way ANOVA. All multiple comparisons were conducted using the Benjamini, Krieger, and Yekutieli two-stage linear step-up procedure to control False Discovery Rate (BKY).

The impact of handler substitution on suckling behaviors (Nutritive suckling, latency to suckle, Cross-sucking, and Displacement-suckle) was assessed for all 14 calves twice a day during their liquid feed intake (n = 28). Binomial scores (cross-sucking and displacement-suckle), Nutritive suckling, ambient temperature (AT, °C), and average daily rainfall (AR, mm) were analyzed with a Kruskal–Wallis test, followed by a post hoc evaluation BKY.

Latency to suckle was analyzed after normalizing the minutes (defining 0% as the smallest and 100% as the largest values in each data set (GraphPad Prism software version 11.0.1 (99)). These data were subsequently analyzed using a One-way ANOVA, incorporating the Geisser-Greenhouse correction to account for potential violations of the sphericity assumption. Post hoc multiple comparisons between sampling periods were performed using Tukey’s test.

Finally, to assess the relationships among environmental variables, physiological stress markers, metabolic indicators, and behavioral modifications, a Spearman rank correlation matrix (two-tailed, 95% CI) was executed. A non-parametric rank correlation was selected due to the non-Gaussian distribution of acute-phase proteins, the non-linear kinetics of neuroendocrine peaks, and the inclusion of dichotomous behavioral variables (0/1). Data used for the correlation matrix included values obtained exclusively from the five calves subjected to blood sampling. For all statistical tests, a value of *p* < 0.05 was considered to indicate statistical significance.

## 3. Results

### 3.1. Physiological and Metabolic Findings

**Figure 2:**
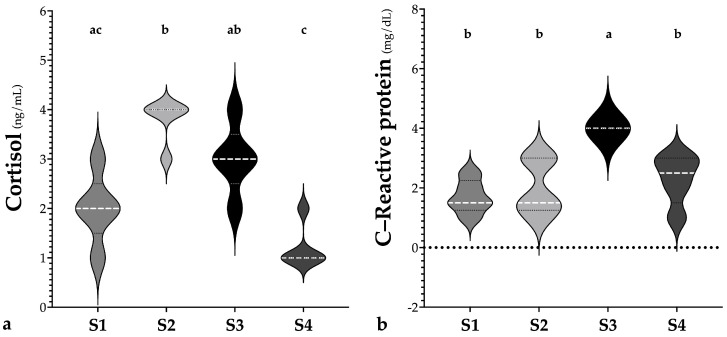
Violin plots showing the distributions of the serum cortisol (ng/mL) (**a**) and C-RP concentrations (mg/Dl) (**b**) of dual-purpose calves (¾ Zebu x ¼ Ho) across four sampling periods influenced by familiar handler substitution. During S1, S3, and S4, the calves were reared in an artificial lactation system with the same handler, while at S2, the handler was replaced. The central white dashed line indicates the median, and the dotted black lines represent the interquartile range (IQR). Different letters indicate significant differences in cortisol (F_(df = 3)_ = 11.64, *p* = 0.0018) and C-RP F_(df = 3)_ = 10.15 *p* = 0.0064). n = 5 per sampling period.

Serum cortisol concentrations varied significantly across the sampling periods (F_(df = 3)_ = 11.64, *p* = 0.0018). Post hoc pairwise comparisons BKY revealed a significant increase in cortisol levels immediately following handler substitution, with a significant difference detected between the baseline (S1) and the acute stress period (S2; Novelty Stress) where it reached its peak concentration (59.38 ± 11.86 ng/mL, q = 0.048, *p* = 0.028). Cortisol concentrations significantly decreased upon the return of the familiar handler (S3), showing a marked drop from the acute stress period (S2) to the final stabilization phase (S4: 11.61 ± 1.93 ng/mL, q = 0.008, *p* = 0.0015). Furthermore, a significant difference was observed between the initial recovery phase (S3: 25.02 ± 8.19 ng/mL) and the final stabilization phase (S4: 11.61 ± 1.93 ng/mL; q = 0.048, *p* = 0.028), confirming the gradual re-establishment of neuroendocrine homeostasis over time (Figure 2a).

Interestingly, exactly 17 days after the peak of hypercortisolism, and even though the familiar handler had already returned, a delayed inflammatory response was observed, with C-reactive protein (C-RP) levels reaching their highest values (100.8 ± 26.73 mg/dL; F_(df = 3)_ = 10.15 *p* = 0.0064). Post hoc multiple comparisons BKY revealed that C-RP levels were significantly higher during S3 compared to both the S1 baseline (q = 0.02, *p* = 0.0048) and the S2 handler substitution period (q = 0.03, *p* = 0.014). The drop in C-RP concentrations in the final stabilization stage (from S3 to S4) was also significant (q = 0.0523, *p* = 0.0373). Finally, it is important to note that in the final stabilization sampling (S4), cortisol levels returned to baseline, and C-RP levels were lower but remained above the threshold (14.40 ± 8.61 mg/dL; Figure 2b).

RR was significantly affected by the sampling periods (F_(2.09, 8.35)_ = 7.20, *p* = 0.015). Multiple comparisons test revealed that RR reached its highest mean value during the handler substitution phase (S2: 37.60 ± 0.75 bpm), which was significantly higher than both S3 (34.20 ± 0.66 bpm, q = 0.046, *p* = 0.018) and the final stabilization phase S4 (31.60 ± 0.98 bpm, q = 0.028, *p* = 0.005). HR showed a statistically significant difference between S2 and S3 (MR diff = 8.5, individual *p* = 0.019). Finally, body temperature showed no significant variations among periods (*p* = 0.378).

### 3.2. Environmental Conditions

Ambient temperature (AT °C) showed significant differences among sampling periods (H_(df = 3)_ = 19.00, *p* = 0.0003). Statistical differences were observed between S1 vs. S2 (*p* = 0.006), S1 vs. S4 (*p* < 0.0001), and between S3 and S4 (*p* = 0.006). Average daily rainfall (AR mm) also varied significantly across the four sampling periods (H_(df = 3)_ = 19.00, *p* = 0.0003). Multiple comparisons indicated that cumulative rainfall was significantly lower during the baseline period (S1) compared to both the return of familiar handler (S3: q = 0.0061, *p* = 0.006) and the final stabilization period (S4: q = 0.0001, *p* < 0.0001). Similarly, rainfall during the handler substitution (S2) was significantly lower than during the S4 (q = 0.0061, *p* = 0.006). Environmental conditions are presented in Table 2.

### 3.3. Behavioral Response to Handler Substitution

**Figure 3:**
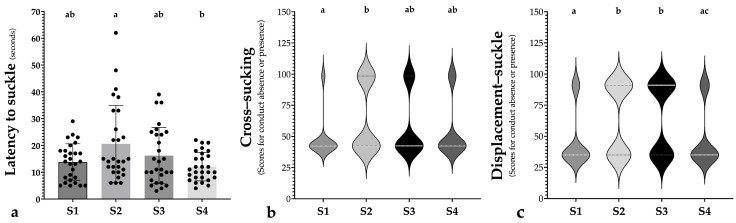
Means (±standard error) plot of the latency to suckle with individual data points shown as black dots (**a**), and violin plots showing the distributions evidence of cross-sucking behavior (**b**), and displacement-suckle (**c**) of dual-purpose calves (¾ Zebu x ¼ Ho) across four sampling periods influenced by familiar handler substitution; n = 14 animals per group with double observation morning and afternoon (n = 28). During S1, S3, and S4, the calves were reared in an artificial lactation system with the same handler, while at S2, the handler was replaced. The white central white dashed line indicates the median, and the dotted black lines represent the interquartile range (IQR). Different letters indicate significant differences (*p* < 0.05).

Across the four sampling days, the mean duration of milk-feeding management for all 14 calves was 26 ± 7.16 min; however, this duration peaked at S2 (36 min), compared with the other sampling periods (S1: 25 min, S3: 19 min, S4: 24 min). Concurrently, the latency to suckle in S2 increased, and was significantly influenced by the sampling period (F_(3, 108)_ = 3.91, *p* = 0.012, R^2^ = 0.098). The maximum latency to suckle was observed during the handler substitution S2 (20.57 ± 2.71 s), which tended to be higher than the baseline (S1: 13.82 ± 1.28 s, *p* = 0.057) and was significantly higher than the final stabilization period (S4: 12.04 ± 0.99 s, *p* = 0.009). Regardless of these management-induced variations, the calves were able to satisfy their feeding behavior; nutritive suckling duration ranged from 1.81 ± 0.08 min (S2) to 2.16 ± 0.13 min (S4) and did not differ significantly among sampling periods (H_(df = 3)_ = 3.99, *p* = 0.26). Showing that once the calf overcomes the initial hesitation, the drive to satisfy hunger overrides the environmental stressor. This pattern demonstrates both the disruption triggered by the handler substitution and the gradual recovery toward homeostatic suckling kinetics after the return of the familiar handler (Table 2; Figure 3a).

Nonetheless, there was evidence of increased abnormal behaviors during and after suckling, such as displacement-suckle of the rubber nipple and cross-sucking. Displacement-suckle during feeding showed significant variation across periods (H_(df = 3)_ = 10.89, *p* = 0.012). Multiple comparisons BKY revealed a significant increase in displacement-suckle occurrences from the baseline period (S1) to both the handler substitution (S2; q = 0.0145, *p* = 0.038) and the initial return of the familiar handler (S3; q = 0.0145, *p* = 0.038). Conversely, behavior frequencies significantly decreased by the final stabilization phase (S4), showing significant differences when compared to both S2 and S3 (q = 0.0150, *p* = 0.028). No significant differences were observed between S1 and S4 (*p* = 0.78) nor between S2 and S3 (*p* > 0.99), indicating that the increased displacement-suckle behaviors observed during the handler’s rotation successfully returned to baseline levels.

Cross-sucking behavior was present during the four observation periods, and even though the overall abnormal behavior presence was not significant (H_(df = 3)_ = 6.4, *p* = 0.093), post hoc multiple comparisons BKY revealed a statistically significant pairwise discovery between S1 and S2 (MR diff = −16.00, q = 0.0294, individual *p* = 0.014) (Figure 3b).

### 3.4. Correlation Analysis of Physiological, Behavioral and Environmental Variables

**Figure 4:**
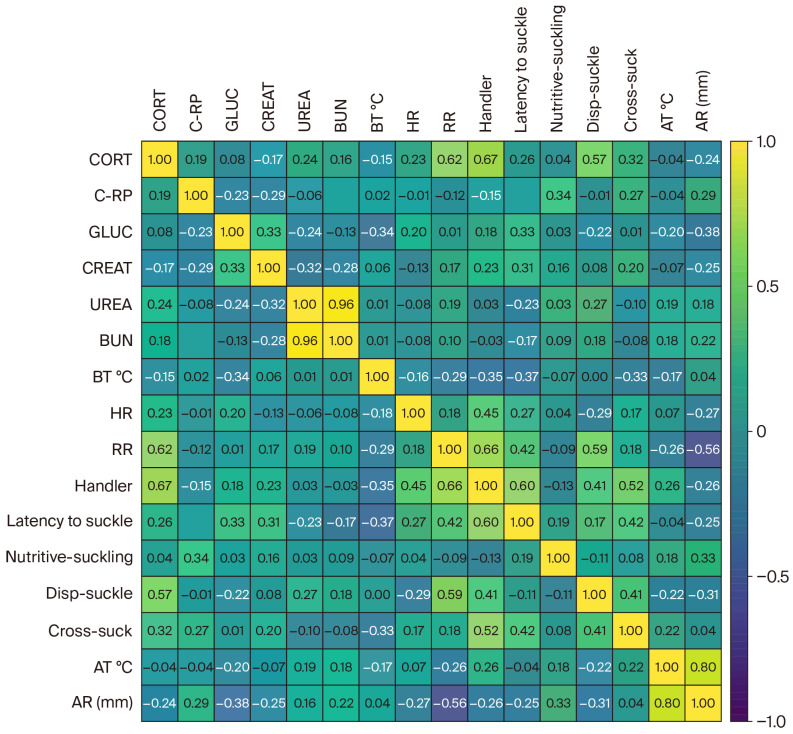
Spearman rank correlation matrix of physiological and metabolic parameters, suckling behaviors, and environmental variables.

The Spearman rank correlation matrix for the five calves sampled revealed critical interactions among sampling periods, physiological indicators, and behavioral modifications (Figure 4). A strong and highly significant positive correlation was identified between handler substitution (Handler) and both serum cortisol levels (r_s_ = 0.67, *p* = 0.001) and respiratory rate (r_s_ = 0.66, *p* = 0.001), linking personnel rotation to heightened physiological stress. Furthermore, cortisol concentrations were also positively correlated with the respiratory rate (r_s_ = 0.62, *p* = 0.004) and with the occurrence of displacement-suckle behavior (Disp-suckle r_s_ = 0.57, *p* = 0.009). The familiar handler substitution also altered feeding patterns, as Handler substitution positively correlated with a prolonged latency to suckle (r_s_ = 0.60, *p* = 0.005), suggesting a delayed response to find a milk feeder, with a simultaneous response in HR (r_s_ = 0.45, *p* = 0.048) and RR (r_s_ = 0.66, *p* = 0.001). Finally, Handler substitution favored an increase in post-feeding abnormal behaviors like cross-sucking (Cross-suck r_s_ = 0.52, *p*= 0.018). No significant correlations were found between C-reactive protein (C-RP), glucose (GLUC), and Nutritive suckling (*p* > 0.05).

Environmentally, the AT °C showed no correlation with cortisol levels (*p* = 0.87) or with the Handler (*p* = 0.27), and its only strong positive correlation was with the average rainfall (r_s_ = 0.80, *p* < 0.0001). Rainfall showed a negative correlation with RR (r_s_ = −0.56, *p* = 0.01). The results indicate that the driver of the neuroendocrine and behavioral changes observed in calves was the handler substitution rather than seasonal climatic variations.

## 4. Discussion

It was found that pre-weaned crossbreed zebu calves under grazing conditions exhibited more negative behavioral, physiological, and metabolic responses to an unfamiliar handler during artificial rearing in the tropics. Regular interactions between handlers and their livestock have a substantial effect on the behavior, stress indicators, welfare, and productivity of farm animals [27,28]; however, less is known about the impact of handler rotation, particularly in calving areas of production units, and even less is known in crossbreed Zebu rearing. Previous research on dairy calves is consistent with the current results, which report negative behavior in response to unfamiliar situations (e.g., the approach of an unfamiliar test person) [2] and to the type of handling provided [29,30]. Alterations to other stress biomarkers have also been identified in calves under unfamiliar situations, such as handling, weaning, and transport, with changes in heart rate [30], leukocyte counts [31], and varying responses in cortisol [32], likely depending on the severity of the stress factors and potentially affecting the productive performance and meat quality in beef calves [33]. Therefore, this study demonstrates that handler substitution triggers a physiological response that begins with acute endocrine shifts and behavioral changes and culminates in a delayed systemic inflammation.

In this study, the most compelling findings to ensure that the calves responded to the handler’s substitution were the increase in plasma cortisol, the latency to suckle, and the onset of cross-sucking as an abnormal behavior at S2. The significant spike in serum cortisol at S2 (59.38 ± 11.86 ng/mL) with a mean difference of 44.56 ng/mL (*p* = 0.028) compared with the first sampling, represented a four-fold increase from basal concentrations observed in S1, and aligns with prior reports identifying environmental and management changes to cause an acute endocrine response and to activate the hypothalamic–pituitary–adrenal (HPA) axis [6,12]. The high correlation between the handler substitution and cortisol levels (CORT r_s_ = 0.67, *p* = 0.001) suggests that in ¾ Zebu x ¼ Holstein calves, the social bond with the handler is strong enough to cause acute stress after separation.

The health consequences of triggering an acute stress response via the HPA axis [34] include impairment of cellular immune functions, thereby increasing susceptibility to illness [35,36], leaving a window of immunological vulnerability. This is consistent with our observations, in which a chronological lag between the hypercortisolism (HYC) peak (S2: 59.38 ± 11.86 ng/mL) and the subsequent post-HYC 17-day peak of C-reactive protein (S3: 100.8 ± 26.73 mg/dL) was evident, even though the familiar handler had already returned. Showing a delayed inflammatory response to HYC, with C-reactive protein (C-RP) levels reaching their highest values (100.8 ± 26.73 mg/dL; *p* = 0.0064). By the 4th sampling period (S4) cortisol levels returned to baseline (11.61 ± 1.93 ng/mL), and even though C-RP levels were lower (14.40 ± 8.61 ng/mL) but still above the healthy calf threshold, restoration indicates a full recovery of homeostasis and can be achieved once the habitual handler returns.

The hypercortisolism observed in this study (59.38 ± 11.86 ng/mL) is a critical finding suggesting that the interruption of handler-calf bonding triggers higher physiological responses and is more stressful than events like rodeo handling, or Burdizzo castration of calves, where reports show cortisol peaks of 21.10 ng/mL [37] and 38.78 ng/mL [38], respectively. Furthermore, the specific spike in cortisol without a corresponding rise in Creatinine at S2 suggests an acute emotional stress response rather than chronic distress or tissue damage [39], confirming that *Bos indicus* crossbreed calves are highly sensitive to the predictability of their environment [26]. The temporary substitution of the familiar handler, despite performing the same tasks, represents a break in predictability, triggering a fight-or-flight response in calves. One of the animals, for instance, showed a cortisol increase from 15.7 ng/mL to 104 ng/mL, demonstrating hyperreactivity and high individual variability in serum cortisol concentrations among animals, suggesting differences in animals’ perception of human-related stress and their coping mechanisms [26,40].

In addition, HPA-axis dysregulation directly affects the overall inflammatory response, as evidenced by the delayed inflammatory response observed in this study. C-reactive protein is considered a non-specific marker of inflammation driven by HPA-axis dysregulation, and in bovines, a serological increase in CRP has been observed during the early stages of infection, even before rectal temperature increases, as well as in response to other physiological factors [41]. Thus, C-reactive protein is a useful marker for assessing stress levels and for early surveillance of disease conditions in cattle [42]. In our study, the C-RP peak (at S3, 17 days after substitution) was followed by a decrease in C-RP upon reinstatement of the familiar handler at S4, (14.40 ± 8.61 mg/dL). The values observed at S4 demonstrate the positive effect of removing the novelty stressor (unfamiliar handler). Similarly, prior reports show a C-RP reversion in both pigs and cows after treatment for specific infections, suggesting that C-RP might be considered a clinical indicator of treatment efficacy [43,44,45]. Furthermore, a potential association between serum C-RP levels and the health conditions of a dairy herd and farm management has been reported, and, consistent with our findings, the authors suggest that C-RP levels could also indicate the farm management environment or serve as a marker of stress [42].

Glucose levels showed no significant correlations or differences across sample periods. Furthermore, in S2, where Hypercortisolism was detected, the glucose level decreased by 5.38% compared with the S1 initial values, while a glucose reduction of 13.36% was observed when C-RP peaked (S3), and it slightly recovered by 2% as C-RP values decreased (S4). The absence of a significant increase in glucose levels despite a cortisol peak at S2 and S3 suggests a high rate of peripheral glucose utilization, which could be associated with the energetic cost of the stress response, due to the offset of cortisol-mediated gluconeogenic effects [46] and heightened maintenance requirements [47]. Furthermore, as the calves’ C-RP was slightly elevated during the cortisol peak, this indicates activation of the innate immune system; cytokines (such as IL-6) that trigger C-RP production also increase glucose uptake by immune cells and can interfere with insulin sensitivity [48]. Finally, our results are also consistent with Davies et al. [49], who reported that chronically stressed calves, when mealtimes are disrupted, have lower glucose levels than non-stressed calves. The stability observed for glucose in this study (88.80 ± 6.99 mg/dL at S1 to 83 ± 3.48 mg/dL at S2 and 76.4 ± 5.35 mg/dL in S3) is consistent with previous studies reporting stress to deplete circulating blood glucose levels due to heightened maintenance requirements [47], or due to the repartitioning of nutrients, where glucose is diverted to the immune system as fast as the liver can produce it [46]. The other metabolic changes in the calves also support secondary stress-related changes. Metabolic markers associated with nitrogen balance, specifically UREA and BUN, showed a near-perfect correlation (r_s_ = 0.96, *p* < 0.0001), validating the technical consistency of the laboratory analysis. Even though both UREA and BUN showed a tendency to increase after hypercortisolism, there were non-significant correlations with the acute cortisol levels (*p* = 0.31 and *p* = 0.50, respectively), which could be associated with a time lag required for glucocorticoid-induced protein catabolism, or the acute emotional stress response rather than chronic stress muscle catabolism [50].

Latency to suckle and cross-sucking were strongly associated with cortisol levels and the handler, with a marked increase in latency to suckle at S2 (20.57 ± 2.71 s) compared with S1 (13.82 ± 1.28 s; *p* = 0.057). This suggests significant situational awareness of the calves. The increased latency to suckle at S2 compared to the other time points could be secondary to the identification of the unfamiliar handler but could also be influenced by the calves’ effects on one another. Boissy et al. [51] reported that increased latency to feed and plasma cortisol in stressed heifers, compared to non-stressed animals, is secondary to a perceived state of stress in conspecifics and that these animals become more fearful as a result [51]. Similarly, in this study, the latency to feeding behavior also reduced in subsequent samplings. In Boissy and colleagues’ experiment, the effect was likely due to habituation after repeated exposures, whereas in this study, the confounding effect of the handler’s reintroduction must be considered. Crucially, this behavioral increase in latency directly extended the overall feeding management duration, which lengthened to 36 min at S2 compared to 25 min at S1. This logistical delay represents an accumulation of auditory, visual, and olfactory stress signals within the six-station calf feeder, which triggered other undesirable behavioral patterns. While the Kruskal–Wallis test for cross-sucking was not fully significant (H_(df = 3)_ = 6.42, *p* = 0.093), the more sensitive post hoc BKY linear step-up procedure detected a significant pairwise discovery between S1 and S2 (C = −16.00, q = 0.029, *p* = 0.014). This indicates that cross-sucking emerged abruptly as an acute, localized coping strategy under the unfamiliar handler’s presence. Additionally, displacement-suckle showed a simultaneous significant increase at S2 and S3 (*p* = 0.014), validating that pathological mouthing behaviors (i.e., cross-sucking) and aggression (i.e., teat displacement) function as a temporary physiological stress-coping mechanism. Nonetheless, these behaviors tended to decrease after the familiar handler was reinstated, highlighting the animal’s strong situational awareness and stress redirection once the environmental predictability was restored.

Our findings demonstrate a clear chronological link between social stress and physiological cost, confirming that a single acute stress event in calves has a lingering biological cost that manifests 17 days later as systemic inflammation. This is consistent with prior reports where cortisol levels above 61 ng/mL were significantly correlated to chronic unresponsive pneumonia 6–7 weeks later [40]. Similarly, other authors have reported that differences of 15 ng/mL among animals correspond to an 80 g/day decrease in average daily gain (ADG) in *Bos indicus* heifers [26]. While previous research using avoidance test methodologies has reported significant findings on the avoidance of unfamiliar or stressful situations, the welfare impact of personnel rotation, a likely common event in livestock systems, should be considered alongside its implications for long-term animal health and welfare management. The findings of this trial highlight the need for further studies assessing the long-term impacts of handler substitution management.

The environmental conditions profile highlights that handler substitution and microclimatic variables exerted concurrent, compounding influences on calf physiology. However, handler substitution exerts a stronger influence than the climatic variables (Figure 4). The endocrine activation of calves was strongly synchronized with autonomic shifts, as evidenced by the significant correlation between cortisol and the respiratory (r_s_ = 0.62; *p* = 0.004), matching the RM One-way ANOVA findings (F_(2.09, 8.35)_ = 7.20, *p* = 0.015, R^2^ = 0.64) where RR peaked significantly at S2 (37.60 ± 0.75 bpm, q = 0.046, *p* = 0.018), suggesting that the acute stress response triggered a compensatory increase in respiratory rate [52] without body temperature changes.

Crucially, ambient temperature (AT °C) showed a significant positive correlation with cortisol levels (r_s_ = 0.45; *p* = 0.05), with the highest temperature registered in S3 (32.4 ± 0.069 °C) and which exhibited a significant decline from the initial recovery phase to the final stabilization phase (S4: 31.16 ± 0.37 °C; Mean diff: 1.24 °C, *p* = 0.021). Concurrently, rainfall (AR mm) showed a significant negative correlation with RR (r_s_ = −0.56; *p* = 0.01), indicating that rainfall events directly reduced respiratory effort, most likely due to decreased ambient heat. This indicates that while the primary driver of acute neuroendocrine disruption and subsequent behavioral modifications at S2 was caregiver substitution rather than seasonal weather shifts, the changing environmental matrix actively shaped the recovery phase (S4). The increasing precipitation and the drop in ambient temperature toward S4 served as microclimatic mitigators that alleviated thermal stress, working in concert with the reinstatement of the familiar handler to facilitate the complete re-establishment of neuroendocrine and behavioral homeostasis.

## 5. Conclusions

Handler substitution during lactation rearing in dual-purpose calves (¾ Zebu x ¼ Ho) is perceived by the animals and elicits an acute stress response. This study suggested that personnel rotation is highly noticeable to calves, with almost immediate physiological responses and behavioral changes that can have prolonged effects on their inflammatory response and thus potentially affect their health and performance. However, if the familiar handler is reinstated, the crossbred Zebu calves may return to basal levels. This highlights the relevance of good farming practices to avoid unnecessary stress if possible.

## Figures and Tables

**Table 1:** Ethogram of dual-purpose calves (¾ Zebu x ¼ Ho) behaviors during artificial lactation.

Behavior	Description
Feeding management	Duration of the feeding behavior starting when entering the six-station calf feeder until the last of the 14 animals finished eating.
Latency to suckle	Time (seconds) from when the animal enters the calf feeding area and finds a milk feeder
Nutritive-suckling	Duration in minutes of activity of the animal with the mouth at the milk feeder with neck movements indicating milk ingestion until the feeding was completed
Cross-sucking	Identification of presence (scored as 1) or absence (scored as 0) of an animal sucking the head or any other body part (mouth of the focal calf, tears, navel, scrotum or udder) of another calf after the lactation period and confined in the in two interconnected pens for solid diet.
Displacement-suckle	Identification of presence (scored as 1) or absence (scored as 0) of a calf that displaced another from a teat

**Table 2:** Longitudinal assessment of physiological, metabolic, and environmental parameters in dual-purpose calves (¾ Zebu x ¼ Ho) across four timepoints (S1, S2, S3, S4).

		Sampling Periods			
	Ref Values [25]	S1	S2	S3	S4
		Physiological and Metabolic Parameters (Mean ± SE)			
Blood urea nitrogen (mg/dL)	9.3–18.6	12.28 ± 1.69 ^a^	13.34 ± 1.13 ^a^	13.88 ± 1.27 ^a^	14.00 ± 1.02 ^a^
Urea (mg/dL)	20–30	26.24 ± 3.60 ^a^	28.50 ± 2.43 ^a^	28.54 ± 3.21 ^a^	29.50 ± 2.38 ^a^
Creatinine (mg/dL)	0.5–1.8	0.9 ± 0.055 ^a^	0.92 ± 0.06 ^a^	0.82 ± 0.06 ^a^	0.84 ± 0.05 ^a^
Glucose (mg/dL)	40–80	88.80 ± 6.99 ^a^	83 ± 3.48 ^a^	76.40 ± 5.35 ^a^	77.60 ± 3.08 ^a^
Cortisol (ng/mL)	0–20 *	14.82 ± 0.41 ^a^	59.38 ± 11.86 ^b^	25.02 ± 8.19 ^ab^	11.61 ± 1.93 ^a^
C-RP (mg/dL)	0–7	4.80 ± 1.20 ^a^	30 ± 18.78 ^a^	100.8 ± 26.73 ^b^	14.40 ± 8.61 ^a^
BT (°C)	38–39.5	38.2 ± 0.18 ^a^	37.78 ± 0.32 ^a^	38.22 ± 0.08 ^a^	38.1 ± 0.18 ^a^
HR (bpm)	80–110	82.2 ± 3.07 ^ab^	88.8 ± 3.66 ^a^	78.4 ± 0.98 ^b^	80.8 ± 0.8 ^ab^
RR (bpm)	15–40	35.0 ± 1.00 ^ab^	37.6 ± 0.74 ^a^	34.2 ± 0.66 ^b^	31.6 ± 0.98 ^b^
Environmental Variables (Mean ± SE)					
Handler		1	2	1	1
AT (°C)		30.57 ^a^	32.3 ^bc^	32.14 ^ab^	32.50 ^c^
AR (mm)		0.91 ^a^	5.64 ^ab^	5.65 ^bc^	11.85 ^c^

Different letters within the same row represents statistical difference (*p* < 0.05). Handler (1 familiar handler; 2 substitute handler). * Cortisol reference values include *Bos indicus* basal cortisol in calmed and excitable steers [26].

## Data Availability

The original contributions presented in this study are included in the article. Further inquiries can be directed to the corresponding authors.

## Abbreviations

The following abbreviations are used in this manuscript:

BUN	Blood Urea Nitrogen
C-RP	C Reactive Protein
GLUC	Glucose
CREAT	Creatinine
CORT	Cortisol
BT	Rectal Temperature
HR	Heart rate
RR	Respiratory rate
AT	Ambient Temperature
AR	Rainfall
